# Severity of Complications after Locking Plate Osteosynthesis in Distal Femur Fractures

**DOI:** 10.3390/jcm13051492

**Published:** 2024-03-05

**Authors:** Roshan Gurung, Alexander Terrill, Gentry White, Markus Windolf, Ladina Hofmann-Fliri, Constantin Dlaska, Michael Schuetz, Devakara R. Epari

**Affiliations:** 1 School of Mechanical, Medical and Process Engineering, Queensland University of Technology, Brisbane City, QLD 4000, Australia; 2 Centre for Biomedical Technologies, Queensland University of Technology, Kelvin Grove, QLD 4059, Australia; 3 School of Clinical Sciences, Faculty of Health, Queensland University of Technology, Kelvin Grove, QLD 4059, Australia; 4 School of Mathematical Sciences, Queensland University of Technology, Brisbane City, QLD 4000, Australia; 5 AO Research Institute Davos, 7270 Davos, Switzerland; 6 AO Innovation Translation Center, AO Foundation, 7270 Davos, Switzerland; 7 The Orthopaedic Research Institute of Queensland, Townsville, QLD 4812, Australia; 8 Jamieson Trauma Institute, Metro North Hospital and Health Services, Herston, QLD 4006, Australia

**Keywords:** risk estimation, distal femoral fractures, plate fixation, locking plate

## Abstract

**Background**: Locked plating for distal femur fractures is widely recommended and used. We systematically reviewed clinical studies assessing the benefits and harms of fracture fixation with locked plates in AO/OTA Type 32 and 33 femur fractures. **Methods**: A comprehensive literature search of PubMed, Embase, Cinahl, Web of Science, and the Cochrane Database was performed. The studies included randomized and non-randomized clinical trials, observational studies, and case series involving patients with distal femur fractures. Studies of other fracture patterns, studies conducted on children, pathological fractures, cadaveric studies, animal models, and those with non-clinical study designs were excluded. **Results**: 53 studies with 1788 patients were found to satisfy the inclusion and exclusion criteria. The most common harms were nonunion (14.8%), malunion (13%), fixation failure (5.3%), infection (3.7%), and symptomatic implant (3.1%). Time to full weight-bearing ranged from 5 to 24 weeks, averaging 12.3 weeks. The average duration of follow-up was 18.18 months, ranging from 0.5 to 108 months. Surgical time ranged between 40 and 540 min, with an average of 141 min. The length of stay in days was 12.7, ranging from 1 to 61. The average plate length was ten holes, ranging from 5 to 20 holes. **Conclusion**: This review aimed to systematically synthesize the available evidence on the risk associated with locked plating osteosynthesis in distal femur fractures. Nonunion is the most common harm and is the primary cause of reoperation. The overall combined risk of a major and critical complication (i.e., requiring reoperation) is approximately 20%.

## 1. Introduction

The development of modern implants and improvements in techniques such as minimally invasive plate osteosynthesis (MIPO) have made surgical fixation state of the art in most distal femoral fractures. Compared to non-surgical treatment, patients treated surgically spent less time in the hospital, returned to activity sooner, had better functional results, and had a lower incidence of nonunion, malunion, and infection [1,2,3]. Modern implants, such as locking plates, offer several new options for managing complex and periprosthetic fractures compared to traditional plating. Unlike traditional plating techniques, where plate compression to bone achieves absolute rigidity, locking plate fixation maintains a certain elasticity to stimulate bone healing [4]. Locking plates do not directly contact the bone, thus preserving the periosteal blood supply [5,6]. Furthermore, when used in osteoporotic bones, locking plate constructs have shown increased fatigue strength, increased ultimate failure load, and improved fixation stability compared to nonlocked implants [7,8,9].

Despite the benefits of locked plating for distal femur fractures, the reported incidence of healing complications like nonunion varies significantly, ranging from 0% [10] to as high as 20% [11,12,13]. The mechanical environment of the fracture (e.g., interfragmentary movement [IFM]) can vary significantly depending on the fixation configuration [14,15], and studies have shown that locked plating constructs are at high risk of becoming too rigid/stiff, thus impairing fracture healing [13,16]. As a result, fixation stiffness can vary (too rigid or highly flexible), affecting callus formation and leading to healing complications [17,18,19].

The primary goal of fixation in distal femur fracture treatment is to minimize disability, restore and maintain limb alignment, preserve blood supply, and encourage early active mobilization. To date, the use of locked plating implants has addressed these goals. However, gaps still exist regarding post-operative early full weight-bearing. While the design of currently used locking plates encourages early and active mobilization through partial weight-bearing, they are contraindicated for early full weight-bearing [20,21,22]. As a result, surgeons remain cautious about early full weight-bearing due to the fear of an early failure of the fixation as the load transmitted to the bone–implant interface is not shared with the bone and can be relatively high [23,24]. Hence, partial toe-touch or touch weight-bearing is often prescribed to patients [25,26]. However, patients do not or cannot adhere to these protocols, compounded by substantial variation in weight-bearing terminology among healthcare professionals [27,28]. Hence, in clinical practice, progressive full weight-bearing is only recommended once the callus is visible on radiographs [10,29].

Recent systematic reviews have focused on comparing complication rates between operative treatments (plating vs. nailing) for distal femur fractures [19,30], including periprosthetic fractures following total knee arthroplasties [31,32]. Studies focusing on locked plating have reviewed contributing factors for nonunion, including patient and intra-operative factors [16,33] and rates of healing difficulties/complications [34]. While these systematic reviews successfully report the frequency of the most commonly occurring clinical complications, such as nonunion and implant failure, a gap exists in reporting the severity of these complications (i.e., the harm). For example, the severity of a nonunion treated conservatively (non-surgical) compared to surgical treatment can change considerably. Combining both parameters (occurrence and severity) allows for a quantitative evaluation of risks associated with locked plating for distal femur fractures. A quantitative risk analysis allows informed decision making on a specific procedure’s benefits, harms, or risks. Hence, to estimate the risk/harms of locking plate osteosynthesis in distal femur fractures, we systematically reviewed all levels of clinical studies, considering additional factors such as time to full weight-bearing and the severity of reported complications.

## 2. Materials and Methods

### 2.1. Search Strategy and Study Selection

The Preferred Reporting Items for Systematic Reviews and Meta-Analyses (PRISMA) guidelines were used. A comprehensive literature search of PubMed, Embase, Cinahl, Web of Science, and the Cochrane Database was performed, including articles from 1990 to 2022 using the search terms defined in Table 1. We included randomized and non-randomized clinical trials, observational studies, and case series involving patients with distal femur fracture/s (AO/OTA Type 32 and 33) requiring osteosynthesis with any type of locking plate. The inclusion and exclusion criteria are shown in Supplementary Data_1 in Tables S1 and S2. Two reviewers independently performed the overall search procedures, including the literature search and data extraction.

### 2.2. Data Extraction

Data were extracted from the articles that fully met the inclusion criteria. The titles and abstracts were scanned for potentially relevant studies, and algorithmic formulas were used to identify duplicates and those outside the inclusion criteria. The full-text versions of the potentially eligible studies were evaluated, and reviewers independently decided on the study’s inclusion. The reference lists of included studies and all literature reviews found in the search results were manually screened for additional articles that met the inclusion criteria. If the methodological procedure described in the full text was considered adequate, it was included. Data from each clinical study were tabulated on a standardized Excel document. Extracted data included author names, publication year, sample size, fracture pattern, patient age and gender, follow-up period, surgical details, and surgical outcome. For commonly reported outcomes, including nonunion, symptomatic implant, malunion, infection, and fixation failure, the severity was considered by categories defined in Table 2. Disagreements on the inclusion of studies were discussed and resolved with a consensus.

### 2.3. Risk Estimation

Risk is the combination of the probability of the occurrence of harm (P_1_) and the severity (P_2_) of the ensuing harm [35,36]. For this review, we defined risks as harms/complications arising from locked plated distal femur fracture treatment, and some commonly occurring harms include nonunion, implant failure, malunion, infection, and symptomatic implant. The P_1_ values were tabulated as a ratio using the total number of reported complications against those populations in which complications were reported. P_2_ is defined as the probability of the different harm severities. The severities (P_2_) of harm were classified as negligible, minor, major, critical, or catastrophic, and only studies that report how all harms were divided into the five different severity classes were included in estimating the P_2_ values. Recognizing that harms can have short-term (e.g., need for surgery or hospitalization) or long-term (e.g., long-term functional impairment) effects, each of these were defined with respect to the health effects, the required management, and the functional outcome. The severity of harm is classified as the severity of its most severe effect (e.g., if harm is asymptomatic, requires surgery, and has no functional deficits, it is classified as major because it requires an invasive intervention). The overall risk was computed using the risk formula described in Appendix A.

## 3. Results

**Figure 1:**
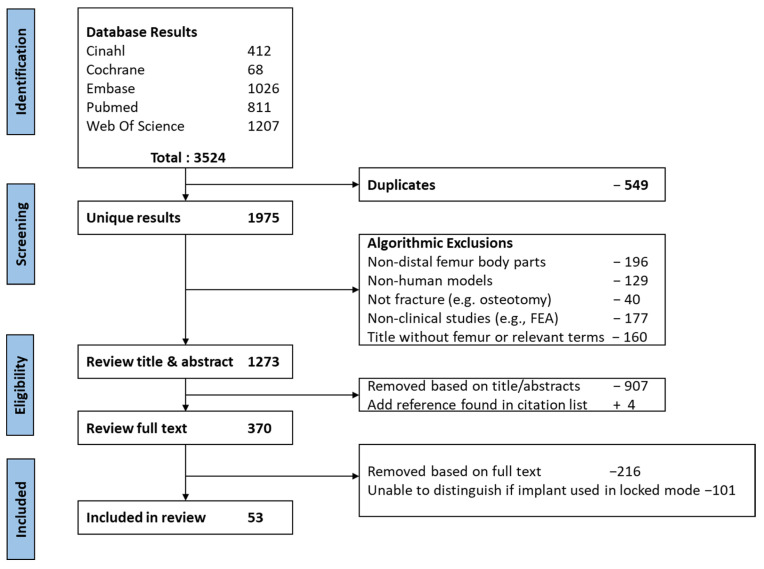
PRISMA (Preferred Reporting Items for Systematic Review and Meta-Analyses) chart illustrating the selection process.

The search strategy and study selection results are demonstrated in the PRISMA flow diagram in Table 1 and Figure 1. The initial search yielded 3524 articles, with 1975 remaining after removing duplicates. Following algorithmic exclusions and abstract screening, 370 studies remained for full-text review. A further 317 studies were removed based on study design, the intervention not being locked plating, or the inability to separate between study group data, leaving 53 eligible studies for inclusion. The results of the 53 studies are summarized in Tables S1 and S2 of Supplementary Data_2.

### 3.1. Findings of Studies

#### 3.1.1. Cohort Characteristics

The 53 papers included 2468 patients with a mean age of 57.4 years, ranging from 16 [37] to 101 years [38]. Of 2468 patients, 680 were excluded due to drop-out or death or excluded by the authors of the paper, leaving 1788 patients for final follow-up. OA/ATO fracture classification, if reported, was as follows: 32A (N_patients_ = 32), 32B (N_patients_ = 25), and 32C (N_patients_ = 9), 33A (N_patients_ = 476), 33B (N_patients_ = 30), 33C (N_patients_ = 589). The literature reported 312 patients (29 papers) with open fractures and 310 patients (28 papers) with periprosthetic fractures.

#### 3.1.2. Surgical Details

The mean duration of the surgery was 141 min, ranging from 40 [39,40,41,42] to 540 min [43]. The average radiation time was 154 s, ranging from 16 [44] to 1800 s [45]. Blood loss averaged 335 mL, ranging from 50 to 3000 mL [43]. Of the 53 papers, only 14 documented the implant size (number of holes), with an average plate length of 10 holes, ranging from 5 to 20 holes [39,40,41,42,46,47,48,49,50,51,52].

#### 3.1.3. Radiological Outcomes

The mean follow-up time was documented in 43 papers, with an average of 18.3 months, ranging from 0.5 [53] to 108 months [54]. The average time to union was 18.88 weeks, ranging from 3 [55] to 104 weeks [56]. The reported union rate without surgical intervention was 85.7%, whereas 9.7% required secondary procedures to promote union [38,43,48,50,57,58,59], and approximately 2.3% had persistent nonunion despite follow-up procedures [57,58]. The time to full weight-bearing was extracted from 12 papers, ranging from 5 [43] to 24 weeks [47,60]. The average hospital stay was 12.7 days, ranging from 1 [60] to 61 days [61].

#### 3.1.4. Post-Operative Complications

Table 3 summarizes the occurrence and the severity of the complication rates, and Table 4 summarizes the overall risk of using locked plating for treating distal femur fractures. The most frequent complication was nonunion, occurring at 14.8%. In the event of nonunion, 68% required surgical management. Fixation failure comprising both the plate and screws occurred at a rate of 5.7%, and most of the time required reoperation (76%). Plate failure occurred at a rate of 2.2%, whereas screw-only failure occurred at a rate of 4.2%. Malunion/malalignment was evident at a rate of 13%, and reoperation was required in approximately 18.8% of the cases. Symptomatic implantation, defined as pain or irritation caused by the implant, occurred at a rate of 3.1%, and approximately 35% required surgical management to remove the implant. The infection rate was 3.7%, and approximately 70% required additional surgical management.

The following additional complications were reported: compartment syndrome at 2.1%, heterotrophic ossification at 1.2%, and distal ischemia/vascular injury at 1.4%. In addition, the following systemic complications were reported: Urinary Tract Infection at 2.3%, chest infection/pneumonia at 1.5%, Deep Vein Thrombosis at 5.7%, Pulmonary Embolism at 2.2%, renal failure or acute kidney injury at 0.7%, gastrointestinal complications at 2.9%.

#### 3.1.5. Clinical Outcomes

Table 5 presents the clinical outcomes of the 53 papers included in this review. Functional outcomes were assessed using a variety of different measures. Knee range of motion (ROM) was assessed in 13 papers, with 296 patients achieving an average knee ROM of 103° (0° to 140°). The mean Hospital for Special Surgery (HSS) score was 78.7, with close to 70% reporting good or excellent outcomes. The mean overall short musculoskeletal functional assessment (SMFA) scores at 6 and 12 months were 46 and 39. The Schatzker and Lambert criteria were assessed in two papers, 45 patients, with more than 50% (24 patients) reporting a good status. Similarly, 45% reported an excellent outcome with the Neer Score.

## 4. Discussion

This review aimed to quantify the risk associated with locked plate osteosynthesis in distal femur fractures. To the best of our knowledge, this is the first review that utilizes a risk assessment framework to report both the occurrence and severity of complications for distal femur fractures treated with locked plating. Existing articles on this topic most commonly report the frequency of complications in terms of a rate, but this does not fully reflect the severity of harm experienced in all cases. Therefore, in this review, we considered additional factors such as the severity of reported complications and time to full weight-bearing along with the occurrence of complications to provide a complete picture of the risks associated with this procedure. This review found that in the distal femur, nonunion is the most commonly occurring complication (14.8%), which is in line with previously reported values of up to 20% [13,18,62] and posed the highest risk for major or higher harm (~10%) (Table 3).

**Figure 2:**
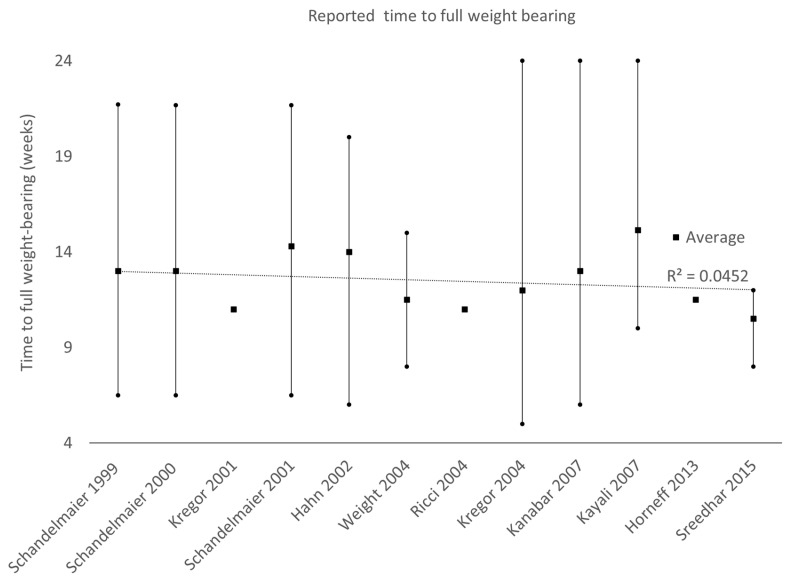
Average time to full weight-bearing over publication date [10,11,40,43,47,48,51,52,60,66,67,68]. Error bars indicate the maximum and minimum time. Over the past two decades, no significant shortening of the patient’s time to full weight-bearing can be observed.

Ultimately, the goal of fracture fixation is to restore function to the injured bone and permit full weight-bearing. Development in implants and improvements in surgical techniques have made internal fixation the treatment of choice in most distal femoral fractures. Despite the improvements, surgically treated patients still undergo an extended period of restricted weight-bearing that constitutes a temporary disability (~12 weeks). Furthermore, there has been no change in the duration of this disability over the past two decades (Figure 2). The temporary disability caused by locked plating is not commonly considered a negative side effect or harm. This is likely because newer osteosynthesis techniques enable early movement and partial weight-bearing, significantly improving patient recovery compared to older methods requiring prolonged immobility. The temporary disability that arises from restricted weight-bearing is due to fear of implant failure, as current implants are not indicated for early full weight-bearing [20,21,22], particularly in patients where healing may be delayed. As a result, surgeons are conservative concerning post-operative weight-bearing and only encourage patients to increase loading once the visible callus is seen on X-rays. Hence, many patients, regardless of injury severity, are subjected to an extended period of temporary disability, which can impact their daily activities. However, the effect and severity of temporary disability can vary among different population groups, such as the young and the elderly. Studies suggest that muscle atrophy from temporary disability caused by restricted weight-bearing can negatively impact overall limb function [63,64,65]. This is particularly true in elderly patients, who are at an increased risk of losing bone mass and strength due to age, bone quality, and activity levels. As a result, elderly patients are more likely to experience complications due to temporary disability.

In contrast, early mobilization and full weight-bearing following a hip fracture enabled through treatment with internal fixation (plates and screws), hemiarthroplasty, and complete hip replacement have improved functional mobility, reduced the length of the hospital stay, and, more importantly, reduced the risk of morbidity and mortality [69,70,71,72,73]. Similarly, early full weight-bearing after ankle fractures has been shown to accelerate the return to work, reduce the hospital stay [74,75], and improve functional outcomes such as ankle range of motion, functional scores, and mental and physical health [76,77]. Hence, decreasing the period of restricted weight-bearing offers an opportunity to improve the care of distal femur fractures. Recent studies have suggested that early full weight-bearing may be possible after distal femur fractures [24,27,78,79]. For example, Kidiyoor and colleagues [78] showed that full weight-bearing is possible as early as 4–5 weeks post-surgery. Additionally, in his study, Poole highlighted the importance of understanding the biomechanical conditions of locked plates to facilitate early full weight-bearing [24]. However, further clinical evidence and studies are needed to fully understand the effects of weight-bearing status on post-operative complications and its benefits to patients.

Weight-bearing is not only important for enabling a return to activities of daily living but has also been shown to be beneficial for timely secondary bone healing and reducing the likelihood of delayed healing and nonunion, with some studies suggesting fracture movements within the first weeks as being critical to healing [80,81,82]. This highlights the complex interaction between weight-bearing, nonunion, and implant failure. The implant failure rate in this study was relatively low (~5%), which in part may be attributed to restricted/partial weight-bearing. However, restrictions in weight-bearing combined with an overly rigid implant [16,17] have been attributed to mechanical causes of nonunion, which, when combined with all causes, amount to ~14%. This highlights the challenges in implant development to achieve a balance in the strength of the implant to support full weight-bearing without implant failure and provide suitable flexibility to the fracture to induce secondary healing.

In this study, we applied a risk assessment framework proposed by Elahi [35] to quantify the harms associated with locked plating of the distal femur. As highlighted earlier, the strength of this approach is the ability to account for the different severities of harm that may arise. In contrast to systematic reviews intended to compare treatment outcomes (i.e., plating versus nailing), where only high-level clinical evidence is often considered to reduce bias, all levels of clinical evidence were included in this study to accurately represent current clinical outcomes. As the published literature is biased toward teaching/research-based hospitals, the results of this review may be skewed and not representative of all clinical settings. This review suggests that temporary disability is important when discussing the effects of early full weight-bearing after fracture fixation. It is important to note that the review did not quantify temporary disability due to a lack of reported data from the articles included. However, based on the severity scale defined in the study, it is considered a major risk and carries the same severity as a requirement of reoperation. Therefore, when deciding on weight-bearing status, it is important to consider the potential risks and benefits and the patient’s individual characteristics, including any comorbidities that may affect the healing process.

## 5. Conclusions

This review has identified a variety of harms associated with locked plates. Nonunion/delayed union is the most common harm and is the primary cause of reoperation. While nonunion is the most reported harm in the literature, an “unrecognized” lesser harm exists, but it is significant in that it affects all patients in the extended period of temporary disability due to delayed full weight-bearing during recovery. Therefore, an opportunity exists to further improve fracture treatment by reducing the period of restricted weight-bearing and temporary disability. However, to do so requires an approach that provides adequate strength for early full weight-bearing without risk of implant failure and suitable flexibility to induce stimulatory micromovement to avoid mechanical nonunion.

## Figures and Tables

**Table 1:** Shows the generic primary search statement used, and minor adjustments were made accordingly to account for the requirements for different databases.

(((plate* OR plati* OR fixat*) AND (lock* OR bridg* OR ((angle OR angul*) AND (stable OR stabili* OR fixed)))) OR “Less Invasive stabilization system” OR “Less invasive stabilization system” OR Axsos OR (non-contact AND bridg*) OR (NCB and Zimmer) OR (LCP AND (plate* OR Synthes)) OR Peri-loc) AND (femur* OR femor*) AND fractur*AND (distal OR supracondyl* OR condyl* OR ((AO OR ASIF OR OTA) AND (33 OR 32))) NOT cadaver AND (english[Language] OR german[Language])	

**Table 2:** Severity definitions for harms following treatment of distal femur fractures according to the Common Terminology Criteria for Adverse Events (CTCAE).

	Negligible	Minor	Major	Critical	Catastrophic
Infection	Exposure, but no clinical manifestation of infection	Managed by use of antibiotics (oral or IV) or outpatient wound management	Requires surgical management, e.g., surgical tissue debridement, implant removal	Systemic life-threatening infection or results in permanent unmanageable functional deficits	Death
Delayed/Nonunion	Asymptomatic with no functional deficit	Does not require revisional surgery	Requires revision surgery or manageable serious permanent functional deficit	Requires revision surgery and results in an unmanageable serious permanent functional deficit	
Symptomatic Implant	Asymptomatic—no intervention required	Pain discomfort related to the implant not requiring surgical management	Pain discomfort related to the implant requires surgical management	Implant results in an unmanageable serious permanent functional deficit and requires surgical management	
Malunion	Asymptomatic with no functional deficit	Malunion not requiring revisional surgery	Malunion that undergoes revisional surgery; significant manageable, functional impacts	Malunion that results in an unmanageable serious permanent functional deficit	
Implant Failure	Does not require revisional surgery	Fixation failure not requiring revisional surgery	Requires revisional surgery or hospitalization. Significant long-term functional impairment	Requires revisional surgery or hospitalization. Requires assistance for mobility and daily living	

**Table 3:** A summary of the occurrence and the severity of the harms identified in this review. The “Total Cases” column represents the number of patients in which the mentioned harm was reported, and “Total Patients” represents the total population in papers that report the harm. The P_1_ column indicates how frequently a given harm occurs. The P_2_ column subdivides each harm into different severities. For example, a major or critical severity would require reoperation, leading to hospitalization, whereas a negligible severity would not require any surgical intervention or hospitalization.

	No. of Articles	Total Cases	Total Patients	Probability of Occurrence (P_1_)	Severity (If Reported) (P_2_)					Risk (P_1_ × P_2_)				
					Negligible	Minor	Major	Critical	Catastrophic	Negligible	Minor	Major	Critical	Catastrophic
Infection	22	17	457	3.7%	0%	30%	40%	30%	0%	0%	1.12%	1.49%	1.12%	0%
Delayed/Nonunion	29	140	944	14.8%	6.3%	25%	50%	18.8%		0.93%	3.71%	7.42%	2.78%	
Symptomatic Implant	55	52	1692	3.1%	54.9%	9.8%	35.3%	0%		1.69%	0.30%	1.08%	0%	
Malunion	25	93	716	13%	25%	56.3%	18.8%	0%		3.25%	7.31%	2.44%	0%	
Implant Failure (Plate and Screw)	34	69	1210	5.7%	0%	24%	76%	0%		0%	1.37%	4.33%	0%	

**Table 4:** A summary of the overall risk of using locked plating for treating distal femur fractures. The results show that when locked plating is used to treat distal femur fractures, 20.66% (major and critical) of the time, surgical intervention is required to address complications.

	Negligible	Minor	Major	Critical	Catastrophic
Overall risk from the use of locking plates in distal femur fractures	5.86%	13.80%	16.76%	3.90%	0%

**Table 5:** Summary of the most reported clinical outcomes.

Knee Range of Motion				
No. of papers (total number of patients)			Mean (range)	
13 (296 patients)			103° (0–140°)	
Neer Score				
No. of papers (No. of patients)	Excellent	Satisfactory	Unsatisfactory	Failure
5 (89 patients)	45%	31%	18%	6%
Hospital of Special Surgery (HSS)				
No. of papers (No. of patients)	Excellent (≥85)	Good (70–84)	Fair (60–69)	Poor (≤60)
4 (72 patients)	33%	36%	12%	7%
Schatzker and Lambert				
No. of papers (No. of patients)	Excellent	Good	Fair	Poor
2 (45 patients)	22%	53%	16%	9%

## Supplementary Materials

The following supporting information can be downloaded at: https://www.mdpi.com/article/10.3390/jcm13051492/s1, Supplementary Data_1: (Table S1: Inclusion criteria; Table S2: Exclusion criteria). Supplementary Data_2: (Table S1: Study Characteristics; Table S2: Reported clinical outcomes).

## Appendix A

### Estimation of Risk

Risk (R) is a combination of occurrence (P_1_) and severity (P_2_).

$$R=P_{1}\times P_{2}$$

Example. A total of 140 nonunion cases were reported in the literature from 29 papers. The total population in the 29 papers where nonunion was reported was 944 patients. Therefore, using the equation below, the probability of the occurrence of nonunion can be estimated to be 14.8%.

(A1) $$P_{1}=\frac{\mathrm{Total}\;\mathrm{reported}\;\mathrm{complication}\;\mathrm{in}\;\mathrm{the}\;29\;\mathrm{articles}\;\left(140\right)}{\mathrm{Total}\;\mathrm{popultion}\;\mathrm{in}\;\mathrm{which}\;\mathrm{complication}\;\mathrm{is}\;\mathrm{reported}\;\left(944\right)}\sim 14.8\%$$

The failure of nonunion can have different consequences from conservative management to surgical management, and P_2_ is the probability of the different severity scales. The severity scales are derived from the literature. Only studies that report how all the harms were divided into the five different severity classes were included.

(A2) P2negligible=number of cases in included studies total sample in included studiesnegligible∑(negligible,minor,major,critical,catastrophic)

Hence, the overall risk of nonunion for the different severities is

(A3) $${\mathrm{Risk}\;\mathrm{of}\;\mathrm{nonunion}}_{\mathrm{negligible}}={\;P}_{1}\times P2_{\mathrm{negligible}}$$

(A4) $${\mathrm{Risk}\;\mathrm{of}\;\mathrm{nonunion}}_{\mathrm{minor}}={\;P}_{1}\times P2_{\mathrm{minor}}$$

(A5) $${\mathrm{Risk}\;\mathrm{of}\;\mathrm{nonunion}}_{\mathrm{major}}={\;P}_{1}\times P2_{\mathrm{major}}$$

(A6) $${\mathrm{Risk}\;\mathrm{of}\;\mathrm{nonunion}}_{\mathrm{critical}}={\;P}_{1}\times P2_{\mathrm{critical}}$$

(A7) $${\mathrm{Risk}\;\mathrm{of}\;\mathrm{nonunion}}_{\mathrm{catastrophic}}={\;P}_{1}\times P2_{\mathrm{catastrophic}}$$

The overall state-of-the-art risk for the other harms is calculated by summing each of the five categories.

Overall risk =3+4+5+6+7
