# Supplementary material for: Severity of Complications after Locking Plate Osteosynthesis in Distal Femur Fractures
Source: J Clin Med. 2024 Mar 5;13(5):1492. doi: 10.3390/jcm13051492 (PMC10934512; doi:10.3390/jcm13051492)
Supplement: Supplementary file 1 [file jcm-13-01492-s001.zip › Supplementary Data_1.pdf]

Supplementary Data 1

Table S1: Inclusion criteria used for this review

	Inclusion criteria	Justification
**Types of studies**	Publication date 1990 (inclusive)-2022<br><br>Studies from any geographical location	Include a large number of papers to capture the current treatment landscape.
	English or German language	Languages of the principal researcher.
	Clinical trials and observational studies (cohort, case series, case-control, prospective, and retrospective)	For the manageability of search results and applicability to the aim of this review.
**Types of participants**	Adults<br><br>Gender: male and female	Treatment landscape to include same target patients with regards to age.
	Receiving standard minimally invasive or open incision surgical treatment for distal femur fractures (including patients with additional injuries)	Validity of reported outcomes and complications.
**Types of outcome measures**	Surgical and implant details, e.g., surgical duration, implant length (screw holes), plate configuration<br><br>Local complications and outcomes<br><br>Systemic complications and outcomes<br><br>Function outcomes: time to weight-bearing, range of motion, and function scores<br><br>Implant complications: symptomatic implant, malunion, nonunion, fixation failure<br><br>Standardized, patient-reported measures of treatment burden / functional capacity.	Applicability of search results to the aim of this review.

Table S2: Exclusion criteria used for this review

	Exclusion criteria	Justification
**Types of studies**	Publication date before 1990	Papers published before this year do not provide a picture of the current treatment landscape.
	Language other than German or English	English and German are the languages of the principal researcher.
	Articles not published in a peer-reviewed journal	Validity of reported outcomes and complications.
	Dissertations /thesis	Does not capture a valid treatment landscape.
	Treatment guidelines documents	
**Types of participants**	Children	Not intended for use in children.
	Participants without distal femur fractures	
	Non-surgical treatment	Applicability of search results to the aim of this review.
	Treatment with methods other than locking plates (e.g., IM nailing) and patients treated with multiple fixation plates (double plating)	
	Distal femur fixation within a drug or tissue trial	
	Participants with bone defects and bone diseases	For the validity of reported outcomes and complications to the aim of this review.
	Surgical procedures outside of the standard minimally invasive and open incision techniques	
**Types of outcome measures**	Economic burden on health services/systems or health professionals and at a society level.	Applicability of search results to the aim of this review.
