# Supplementary material for: Severity of Complications after Locking Plate Osteosynthesis in Distal Femur Fractures
Source: J Clin Med. 2024 Mar 5;13(5):1492. doi: 10.3390/jcm13051492 (PMC10934512; doi:10.3390/jcm13051492)
Supplement: Supplementary file 1 [file jcm-13-01492-s001.zip › Supplementary Data_2.pdf]

Supplementary Data_2

Table S1: Study characteristics of the articles reported in this review. Blank cells indicate that no data was provided, while "0" indicate no outcomes were observed: PPF - Periprosthetic Fractures; St - Surgical time (mins); Rt - Radiation time (secs); HS - Hospital Stay (days); Is - Implant Size (number of screw holes).

Paper	No. Pts (Total available)	Mean age	Follow up details				Fracture Characteristics								Surgical Details			
			RIP	Other	No. Pts (available for follow-up)	Mean Follow up time	Open	PPF	32			33			St	Rt	HS	IS
									A	B	C	A	B	C				
Zhang et al. [1]	31	46.2	0	0	31	14.2	0	0	0	0	0	0	0	31	79.1		10	
Hanschen et al. [2]	12	62.9	0	0	12	12	3	1	0	0	0	5	1	5	134.1	188.9		9.3
Kregor et al. [3]	66	49.0	0	0	66	9			0	0	0	33	0	33	209			
Syed et al. [4]	29	65.3	3	1	25	18	4		3	0	0	4	2	9				
Lill et al. [5]	20	44.8	0	0	20	53	0	0	0	0	0	12	1	7				
Tank et al. [6]	21	61.4	0	0	21	11.8	6		0	0	0	9	0	12				
Kanabar et al. [7]	17	65.3	1	0	16	12	3		0	0	0	8	0	9				9
Weight et al. [8]	26	44.0	2	3	21	18	6	2	0	0	0	7	0	15				
Schutz et al. [9]	112	54.0	8	8	96	13.7	34	12	12	14	5	27	6	52				10
Canadian Orthopaedic Trauma Society et al. [10]	28	0.0	2	4	22				0	0	0							
Markmiller et al. [11]	16	57.2	0	0	16			6	0	0	0	10	0	10	155			9
Buckley et al. [12]	13	55.8	0	0	13		0		0	0	0	9	0	4				
Fankhauser et al. [13]	29	57.0	2	0	27	20	14		0	0	0	10	0	19			22	
Schandelmaier et al. [14]	29	54.0	0	0	29	6	8	2	0	3	0	8	1	17	131	142		9
Kayali et al. [15]	26	49.0	0	0	26	25.8	7		0	0	0	14	0	13			12.6	
Gavaskar et al. [16]	20	73.0	0	0	20	39		19										
Kiluçoglu et al. [17]	16	76.7	0	7	9	21		9										
Demirtas et al. [18]	18	36.0	0	13	5	31.3	7	0	0	0	0	3	5	7				
Park et al. [19]	41	75.0	0	20	21	30.5	0	21	0	0	0	21	0	0				
Kolb et al. [20]	50	49.0		19	31	29	5		0	0	0	7	0	24	135	140		10
Kolb [21]	23	77.0	3	1	19	46	0	19						19	127	135		11

Aldrian [22]	86	75.6	0	38	48	12												
Batista [23]	32	47.3	0	4	28	21.3	15					7		22			7.8	
Horneff [24]	63	68.3	0	35	28	6		28							155.3			
Smith [25]	52	74.0	2	0	50	6												
Southeast Fracture Consortium et al. [26]	339	56.0	0	154	185		78	30										
Ricci et al. [27]	25	41.0	2	5	18	30	10							19				
Toole et al. [28]	24	79.5	4	1	19	11	0	24				24			110.2			10
Althausen et al. [29]	11	82.8		6	5	2.7		5				5			135			
Hierholzer et al. [30]	115	54.0		59	56	15	31					21		35				
Hoffmann et al. [31]	54	73.2	0	19	35	22	2	36				36					6.9	
Apostolou et al. [32]	19	60.4	3	9	7	16	2	3				4		4				
Christ et al. [33]	40	76.0		33	7	21.4		7										
Gao et al. [34]	36	54.7		17	19	23.4						19			79.7			
Liu et al. [35]	189	49.0	0	104	85	27			4	3	1	29	7	40				
Wong et al. [36]	16	75.0	0	0	16	23						8	3	5				
Srinivas et al. [37]	40	67.0	3	0	37	12		14	1 3	2	3	15		4				
Batchelor et al. [38]	27	64.7	0	8	19	30									118.4		11.7	
Kregor et al. [39]	119	52.0	3	14	102	14	35					44		59	183			11
Ehlinger et al. [40]	16	81.0	0	0	16	36.5	0	16										
Schütz et al. [41]	62	52.4	8	2	52		21					22		44	112.6	220.8		9
Fulkerson et al. [42]	26	69.4	0	2	24	27		24							90			
Schandelmaier et al. [43]	29	54.0	0	0	29	6	8			3		8	1	17	131	142		10
Wick et al. [44]	18	80.3	0	9	9	18.2						9			102.3	43	12.7	
Hahn et al. [45]	10	49.7	1	1	8		3	1				2		8				
Schütz et al. [46]	112	54.0	8	7	97	13.7												
Schandelmaier et al. [47]	51	53.0	2	1	48		10	6				10	2	36	137	127		9
Sreedhar et al. [48]	30	37.0	0	0	30													
Abdelmonem et al. [49]	20	52.8	0	0	20							14		6				
Paulsson et al. [50] - FWB group	11	52.8	1	1	9			4				5	1	1	83		14.6	
Paulsson et al. [50] - PWB group	21	79.2	3	2	16			8				7		3	104		14.3	
Jang et al. [51] – Group 1	33	81.3	0	0	33			1							149			10.9
Jang et al. [51] – Group 2	32	51.0	0	0	32			4							165			11.3

Kanakaris et al. [52]	19	77.4	0	0	19			8							100.4		20.6	
Jain et al. [53]	38	40.8	2	10	26	11.6												

Table S2: Total number of reported outcomes observed for each of the 53 publications. Blank cells indicate that no data was provided for that outcome, while "0" indicate no outcomes were observed/reported. In - Infection; FF - Fixation Failure; D/NU Delayed/Nonunion; SI - Symptomatic Implant; Mal – Malunion; CS - compartment syndrome; HO - heterotrophic ossification; VI - vascular injury; UTI - Urinary Tract Infection; Pn - pneumonia; DVT – Deep Vein Thrombosis; PE - pulmonary embolism; RF – Renal Failure; GI - gastrointestinal complications.

Paper	Outcome Details		Complications					Local Complications			Systemic Complications					
	Time to union (weeks)	Time to Full weight-bearing (weeks)	In	FF	D/NU	SI	Mal	CS	HO	VI	UTI	Pn	DVT	PE	RF	GI
Zhang et al. [1]			0	0	1	0										
Hanschen et al. [2]			0		4	0		0								
Kregor et al. [3]		11	2	0		0	3									
Syed et al. [4]	14.3		1	0	3	0	1	1	0	0	0	0	0	1	0	0
Lill et al. [5]				1		0	9									
Tank et al. [6]				3		0										
Kanabar et al. [7]	17	13	0	0	3	4	2	0	0	0	2	2	0	0	0	0
Weight et al. [8]	13	11.5	0	0	0	4	3									
Schutz et al. [9]					9	0			1		0	0	1	1	0	1
Canadian Orthopaedic Trauma Society et al. [10]	32					2							1	1		
Markmiller et al. [11]	13.8		0			0	3									
Buckley et al. [12]						0	5									
Fankhauser et al. [13]	12		0			0				0			0	0		
Schandelmaier et al. [14]	13.4	13		2		0	7		1				1	1		
Kayali et al. [15]		15.1	3	2	0	0	3									
Gavaskar et al. [16]	21				3	0									1	
Kiluçoglu et al. [17]	16.9					0										
Demirtas et al. [18]	25.7			1	5	0	4									

Park et al. [19]	15.6		0	0		0	1									
Kolb et al. [20]	12					0	5									
Kolb [21]	14		0		2	0	1						1			
Aldrian [22]			1	3	7	0	1									
Batista [23]			0	4	2	0	8									
Horneff [24]	11.5	11.5				0										
Smith [25]				3	1	0	2						9	1		
Southeast Fracture Consortium et al. [26]				6	41	0										
Ricci et al. [27]		11	0		0	0	1									
Toole et al. [28]			0	1	0	0	0									
Althausen et al. [29]						0	5									
Hierholzer et al. [30]			5		6	0										
Hoffmann et al. [31]					8	0	5				1			1		
Apostolou et al. [32]	12.5		0	2		0										
Christ et al. [33]			0		1	0					1		3			
Gao et al. [34]				1	7	0										
Liu et al. [35]	15			2		0	4		1							
Wong et al. [36]	30		0	2		0										
Srinivas et al. [37]	17.3			2		0										
Batchelor et al. [38]				5	6	3	5									
Kregor et al. [39]		12		5		0	6		0					1		
Ehlinger et al. [40]	10			0	1	0										
Schütz et al. [41]				3	8	0										
Fulkerson et al. [42]	6.2			1	5	0										
Schandelmaier et al. [43]		13				0			1				1	1		
Wick et al. [44]						0				1						
Hahn et al. [45]		14				0										
Schütz et al. [46]				4		0			1							
Schandelmaier et al. [47]	14.3	14.3		4	3	0			1				2	2		
Sreedhar et al. [48]	20	10.5				0										
Abdelmonem et al. [49]	15		2	0		0	7									
Paulsson et al. [50] - FWB group					1	0										
Paulsson et al. [50] - PWB group			1			0										
Jang et al. [51] – Group 1	32.1				2	0										

Jang et al. [51] – Group 2	46.4				3	0										
Kanakaris et al. [52]						0										
Jain et al. [53]	38.5		2	2	8	0										

References

[1] Zhang R, Yang Z, Chen K, Guo X, Huang W. Clinical effect of three-dimensional printing assisted less invasive stabilization system (LISS) to treat femoral intercondylar fracture. Int J Clin Exp Med 2018;11:10809–14.

[2] Hanschen M, Biberthaler P. Re: Mono- versus polyaxial locking plates in distal femur fractures: a prospective randomized multicentre clinical trial. Int Orthop 2014;38:1751–2. https://doi.org/10.1007/s00264-014-2385-z.

[3] Kregor PJ, Hughes JL, Cole PA. Fixation of distal femoral fractures above total knee arthroplasty utilizing the Less Invasive Stabilization System (L.I.S.S.). Injury 2001;32:64–75. https://doi.org/10.1016/s0020-1383(01)00185-1.

[4] Syed AA, Agarwal M, Giannoudis P V., Matthews SJEE, Smith RM. Distal femoral fractures: long-term outcome following stabilisation with the LISS. Injury 2004;35:599–607. https://doi.org/10.1016/S0020-1383(03)00247-X.

[5] Lill M, Attal R, Rudisch A, Wick MC, Blauth M, Lutz M. Does MIPO of fractures of the distal femur result in more rotational malalignment than ORIF? A retrospective study. Eur J Trauma Emerg Surg 2016;42:733–40. https://doi.org/10.1007/s00068-015-0595-8.

[6] Tank JC, Schneider PS, Davis E, Galpin M, Prasarn ML, Choo AM, et al. Early mechanical failures of the synthes variable angle locking distal femur plate. J Orthop Trauma 2016;30:e7–11. https://doi.org/10.1097/BOT.0000000000000391.

[7] Kanabar P, Kumar V, Owen PJ, Rushton N. Less invasive stabilisation system plating for distal femoral fractures. J Orthop Surg (Hong Kong) 2007;15:299–302. https://doi.org/10.1177/230949900701500311.

[8] Weight M, Collinge C. Early results of the Less Invasive Stabilization System for mechanically unstable fractures of the distal femur (AO/OTA types A2, A3, C2, and C3). J Orthop Trauma 2004;18:503–8. https://doi.org/10.1097/00005131-200409000-00005.

[9] Schütz M, Müller M, Krettek C, Höntzsch D, Regazzoni P, Ganz R, et al. Minimally invasive fracture stabilization of distal femoral fractures with the LISS: A prospective multicenter study results of a clinical study with special emphasis on difficult cases. Injury 2001;32:48–54. https://doi.org/10.1016/S0020-1383(01)00183-8.

[10] Canadian Orthopaedic Trauma Society. Are locking constructs in distal femoral fractures always best? A prospective multicenter randomized controlled trial comparing the less invasive stabilization system with the minimally invasive dynamic condylar screw system. J Orthop Trauma 2016;30:e1–6. https://doi.org/10.1097/BOT.0000000000000450.

[11] Markmiller M, Konrad G, Südkamp N. Femur-LISS and distal femoral nail for fixation of distal femoral fractures: Are there differences in outcome and complications? Clin Orthop Relat Res 2004;426:252–7. https://doi.org/10.1097/01.blo.0000141935.86481.ba.

[12] Buckley R, Mohanty K, Malish D, Buckley R, Mohanty K, Malish D. Lower limb malrotation following MIPO technique of distal femoral and proximal tibial fractures. Injury 2011;42:194–9. https://doi.org/10.1016/j.injury.2010.08.024.

[13] Fankhauser F, Gruber G, Schippinger G, Boldin C, Hofer HP, Grechenig W, et al. Minimal-invasive treatment of distal femoral fractures with the LISS (Less Invasive Stabilization System) - A prospective study of 30 fractures with a follow up of 20 months. ACTA Orthop Scand 2004;75:56–60. https://doi.org/10.1080/00016470410001708110.

[14] Schandelmaier P, Krettek C, Miclau T, Stephan C, Konemann B, Tscherne H. Stabilization of distal femoral fractures using the LISS. Tech Orthop 1999;14:230–46. https://doi.org/10.1097/00013611-199909000-00009.

[15] Kayali C, Agus H, Turgut A. Successful results of minimally invasive surgery for comminuted supracondylar femoral fractures with LISS: comparative study of multiply injured and isolated femoral fractures. J Orthop Sci 2007;12:458–65. https://doi.org/10.1007/s00776-007-1156-8.

[16] Gavaskar AS, Tummala NC, Subramanian M. The outcome and complications of the locked plating management for the periprosthetic distal femur fractures after a total knee arthroplasty. Clin Orthop Surg 2013;5:124–8. https://doi.org/10.4055/cios.2013.5.2.124.

[17] Kiluçoğlu OI, Akgül T, Sağlam Y, Yazicioğlu O. Comparison of locked plating and intramedullary nailing for periprosthetic supracondylar femur fractures after knee arthroplasty. Acta Orthop Belg 2013;79:417–21.

[18] Demirtas A, Azboy I, Özkul E, Gem M, Alemdar C. Comparison of retrograde intramedullary nailing and bridge plating in the treatment of extra-articular fractures of the distal femur. Acta Orthop Traumatol Turc 2014;48:521–6. https://doi.org/10.3944/AOTT.2014.14.0004.

[19] Park J, Lee J, Lee JH. Comparison of retrograde nailing and minimally invasive plating for treatment of periprosthetic supracondylar femur fractures (OTA 33-A) above total knee arthroplasty. Arch Orthop Trauma Surg 2016;136:331–8. https://doi.org/10.1007/s00402-015-2374-8.

[20] Kolb W, Guhlmann H, Windisch C, Marx F, Kolb K, Koller H. Fixation of distal femoral fractures with the less invasive stabilization system: A minimally invasive treatment with locked fixed-angle screws. J Trauma Inj Infect Crit Care 2008;65:1425–34. https://doi.org/10.1097/TA.0b013e318166d24a.

[21] Kolb W, Guhlmann H, Windisch C, Marx F, Koller H, Kolb K. Fixation of periprosthetic femur fractures above total knee arthroplasty with the less invasive stabilization system: A midterm follow-up study. J Trauma - Inj Infect Crit Care 2010;69:670–6. https://doi.org/10.1097/TA.0b013e3181c9ba3b.

[22] Aldrian S, Schuster R, Haas N, Erhart J, Strickner M, Blutsch B, et al. Fixation of supracondylar femoral fractures following total knee arthroplasty: is there any difference comparing angular stable plate fixation versus rigid interlocking nail fixation? Arch Orthop Trauma Surg 2013;133:921–7. https://doi.org/10.1007/s00402-013-1730-9.

[23] Batista BB, Salim R, Paccola CAJ, Kfuri Junior M. Internal fixators: a safe option for managing distal femur fractures? Acta Ortopédica Bras 2014;22:159–62. https://doi.org/10.1590/1413-78522014220300509.

[24] Horneff JG, Scolaro JA, Jafari SM, Mirza A, Parvizi J, Mehta S. Intramedullary Nailing Versus Locked Plate for Treating Supracondylar Periprosthetic Femur Fractures. Orthopedics 2013;36:e561--e566. https://doi.org/10.3928/01477447-20130426-16.

[25] Smith WR, Stoneback JW, Morgan SJ, Stahel PF. Is immediate weight bearing safe for periprosthetic distal femur fractures treated by locked plating? A feasibility study in 52 consecutive patients. Patient Saf Surg 2016;10:26. https://doi.org/10.1186/s13037-016-0114-9.

[26] Southeast Fracture Consortium. LCP versus LISS in the treatment of open and closed distal femur fractures: Does it make a difference? J Orthop Trauma 2016;30:e212–6. https://doi.org/10.1097/BOT.0000000000000507.

[27] Ricci AR, Yue JJ, Taffet R, Catalano JB, DeFalco RA, Wilkens KJ. Less Invasive Stabilization System for treatment of distal femur fractures. Am J Orthop (Belle Mead NJ) 2004;33:250–5.

[28] O'Toole R V, Gobezie R, Hwang R, Chandler AR, Smith RM, Estok DM, et al. Low complication rate of LISS for femur fractures adjacent to stable hip or knee arthroplasty. Clin Orthop Relat Res 2006:203–10. https://doi.org/10.1097/01.blo.0000223987.59702.17.

[29] Althausen PL, Lee MA, Finkemeier CG, Meehan JP, Rodrigo JJ. Operative stabilization of supracondylar femur fractures above total knee arthroplasty: A comparison of four treatment methods. J Arthroplasty 2003;18:834–9. https://doi.org/10.1016/S0883-5403(03)00339-5.

[30] Hierholzer C, Rüden C, Pötzel T, Woltmann A, Bühren V. Outcome analysis of retrograde nailing and less invasive stabilization system in distal femoral fractures: A retrospective analysis. Indian J Orthop 2011;45:243. https://doi.org/10.4103/0019-5413.80043.

[31] Hoffmann MF, Jones CB, Sietsema DL, Koenig SJ, Tornetta P. Outcome of periprosthetic distal femoral fractures following knee arthroplasty. Injury 2012;43:1084–9. https://doi.org/10.1016/j.injury.2012.01.025.

[32] Apostolou CD, Papavasiliou A V., Aslam N, Handley RC, Willett KM. Preliminary results and technical aspects following stabilisation of fractures around the knee with liss. Inj Extra 2005;36:529–36. https://doi.org/10.1016/j.injury.2005.02.046.

[33] Christ AB, Chawla H, Gausden EB, Villa JC, Wellman DS, Lorich DG, et al. Radiographic and Clinical Outcomes of Periprosthetic Distal Femur Fractures Treated With Open Reduction Internal Fixation. J Orthop Trauma 2018;32:515–20. https://doi.org/10.1097/BOT.0000000000001265.

[34] Gao K, Gao W, Huang J, Li H, Li F, Tao J, et al. Retrograde Nailing versus Locked Plating of Extra-Articular Distal Femoral Fractures: Comparison of 36 Cases. Med Princ Pract 2013;22:161–6. https://doi.org/10.1159/000342664.

[35] Liu F, Tao R, Cao Y, Wang Y, Zhou Z, Wang H, et al. The role of LISS (less invasive stabilisation system) in the treatment of peri-knee fractures. Injury 2009;40:1187–94. https://doi.org/10.1016/j.injury.2009.03.012.

[36] Wong MK, Leung F, Chow SP. Treatment of distal femoral fractures in the elderly using a less-invasive plating technique. Int Orthop 2005;29:117–20. https://doi.org/10.1007/s00264-004-0609-3.

[37] Srinivas S, Maru M, Lennox C. Treatment of distal femoral fractures using the Less Invasive Stabilisation System (LISS) plates. J Orthop 2008;5:6p-6p.

[38] Batchelor E, Heal C, Haladyn JK, Drobetz H. Treatment of distal femur fractures in a regional Australian hospital. World J Orthop 2014;5:379–85. https://doi.org/10.5312/wjo.v5.i3.379.

[39] Kregor PJ, Stannard JA, Zlowodzki M, Cole PA. Treatment of distal femur fractures using the Less Invasive Stabilization System: Surgical experience and early clinical results in 103 fractures. J Orthop Trauma 2004;18:509–20. https://doi.org/10.1097/00005131-200409000-00006.

[40] Ehlinger M, Adam P, Abane L, Rahme M, Moor BK, Arlettaz Y, et al. Treatment of periprosthetic femoral fractures of the knee. Knee Surgery, Sport Traumatol Arthrosc 2011;19:1473–8. https://doi.org/10.1007/s00167-011-1480-6.

[41] Schütz M, Müller M, Regazzoni P, Höntzsch D, Krettek C, Van der Werken C, et al. Use of the Less Invasive Stabilization System (LISS) in patients with distal femoral (AO33) fractures: A

prospective multicenter study. Arch Orthop Trauma Surg 2005;125:102–8. https://doi.org/10.1007/s00402-004-0779-x.

[42] Fulkerson E, Tejwani N, Stuchin S, Egol K. Management of periprosthetic femur fractures with a first generation locking plate. Injury 2007;38:965–72. https://doi.org/10.1016/j.injury.2007.02.026.

[43] Schandelmaier P, Stephan C, Tscherne H. Die kiSS-Platte bei distalen Oberschenkeifrakturen 2000;3:75–8. https://doi.org/10.1007/BF02949871.

[44] Wick M, Müller EJ, Kutscha-Lissberg F, Hopf F, Muhr G. Die operative versorgung suprakondylärer femurfrakturen bei liegender knieendoprothese - "Less invasive stabilization system" (LISS) oder retrograder marknagel? Probleme in der anwendung minimal-invasiver verfahren. Unfallchirurg 2004;107:181–8. https://doi.org/10.1007/s00113-003-0723-5.

[45] Hahn U, Prokop A, Jubel A, Isenberg J, Rehm KE. LISS versus condylar plate. Kongressband Dtsch Gesellschaft fur Chir Kongress 2002;119:498–504.

[46] Schütz M, Haas NP, Schutz M, Haas NP. LISS--internal plate fixator. Kongressbd Dtsch Ges Chir Kongr 2001;118:375–9. https://doi.org/10.1007/978-3-642-56458-1_123.

[47] Schandelmaier P, Partenheimer A, Koenemann B, Grün OA, Krettek C. Distal femoral fractures and LISS stabilization. Injury 2001;32:55–63. https://doi.org/10.1016/s0020-1383(01)00184-x.

[48] Sreedhar M, M E L, B A, Prashanth P, Swaroopa S, Madhav V. Functional and Radiological Outcome of Distal Femoral Fractures Treated With Locking Compression Plate. J Evol Med Dent Sci 2015;4:6664–71. https://doi.org/10.14260/jemds/2015/964.

[49] Abdelmonem AH, Saber AY, El Sagheir M, El-Malky A. Evaluation of the Results of Minimally Invasive Plate Osteosynthesis Using a Locking Plate in the Treatment of Distal Femur Fractures. Cureus 2022;14. https://doi.org/10.7759/cureus.23617.

[50] Paulsson M, Ekholm C, Jonsson E, Geijer M, Rolfson O. Immediate Full Weight-Bearing Versus Partial Weight-Bearing After Plate Fixation of Distal Femur Fractures in Elderly Patients. A Randomized Controlled Trial. Geriatr Orthop Surg Rehabil 2021;12:1–14. https://doi.org/10.1177/21514593211055889.

[51] Jang JH, Rhee SJ, Jun S Bin, Choi YY. Scattering and clustering the proximal screw construct in unilateral locking plate osteosynthesis of distal femoral fractures. Arch Orthop Trauma Surg 2021. https://doi.org/10.1007/s00402-021-03912-1.

[52] Kanakaris NK, Obakponovwe O, Krkovic M, Costa ML, Shaw D, Mohanty KR, et al. Fixation of periprosthetic or osteoporotic distal femoral fractures with locking plates: a pilot randomised controlled trial. Int Orthop 2018;(no pagina:1193–204. https://doi.org/10.1007/s00264-018-4061-1.

[53] Jain D, Arora R, Garg R, Mahindra P, Selhi HS. Functional outcome of open distal femoral fractures managed with lateral locking plates. Int Orthop 2020;44:725–33. https://doi.org/10.1007/s00264-019-04347-5.
